# The Claustrum Sign in Febrile Infection-Related Epilepsy Syndrome (FIRES)

**DOI:** 10.5334/jbsr.3142

**Published:** 2023-06-29

**Authors:** Kelly Di Dier, Lucas Dekesel, Sven Dekeyzer

**Affiliations:** 1 UZ Ghent, BE

**Keywords:** claustrum sign, FIRES, NORSE, magnetic resonance imaging, neuroradiology

## Abstract

**Teaching Point:** Bilateral mesiotemporal edema and the claustrum sign can be seen in *febrile infection-related epilepsy syndrome* (FIRES) and *new onset refractory status epilepticus* (NORSE) but are neither diagnostic nor pathognomonic for these entities.

## Case History

**Figure 1:**
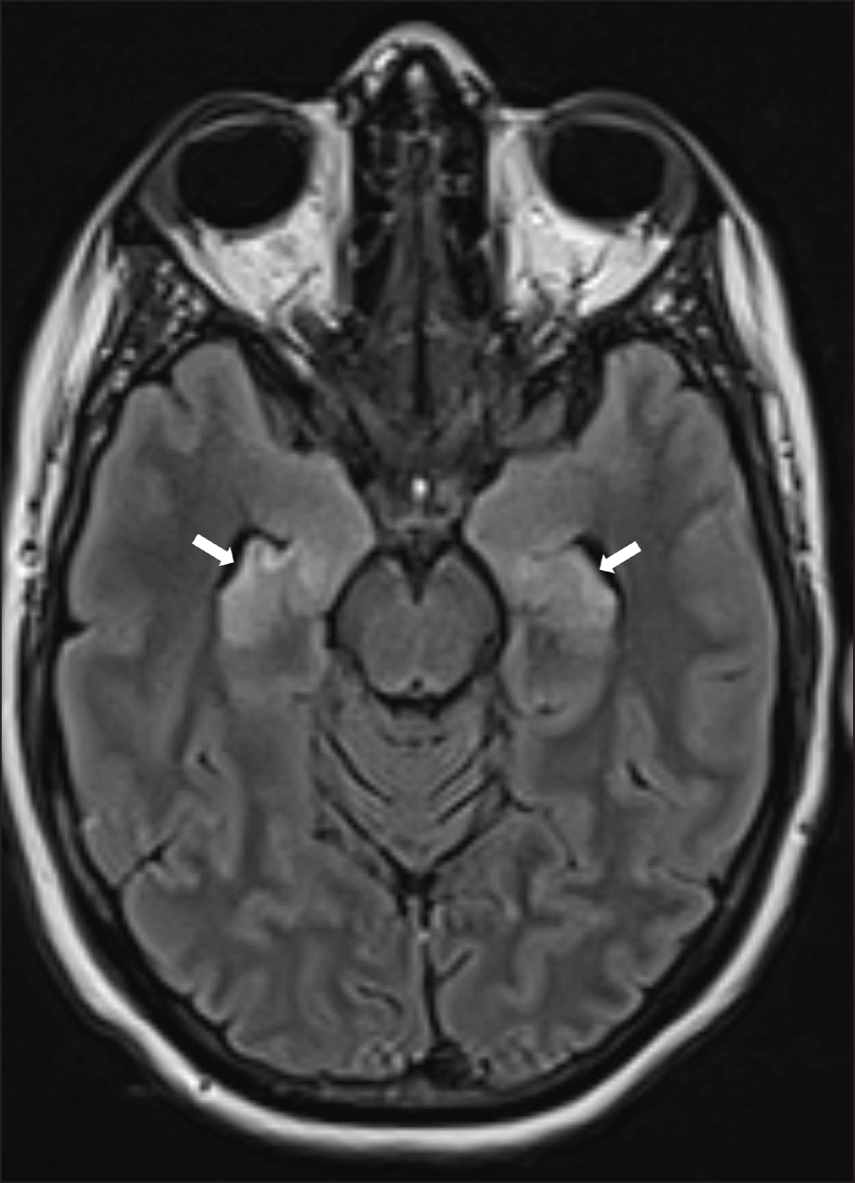


**Figure 2:**
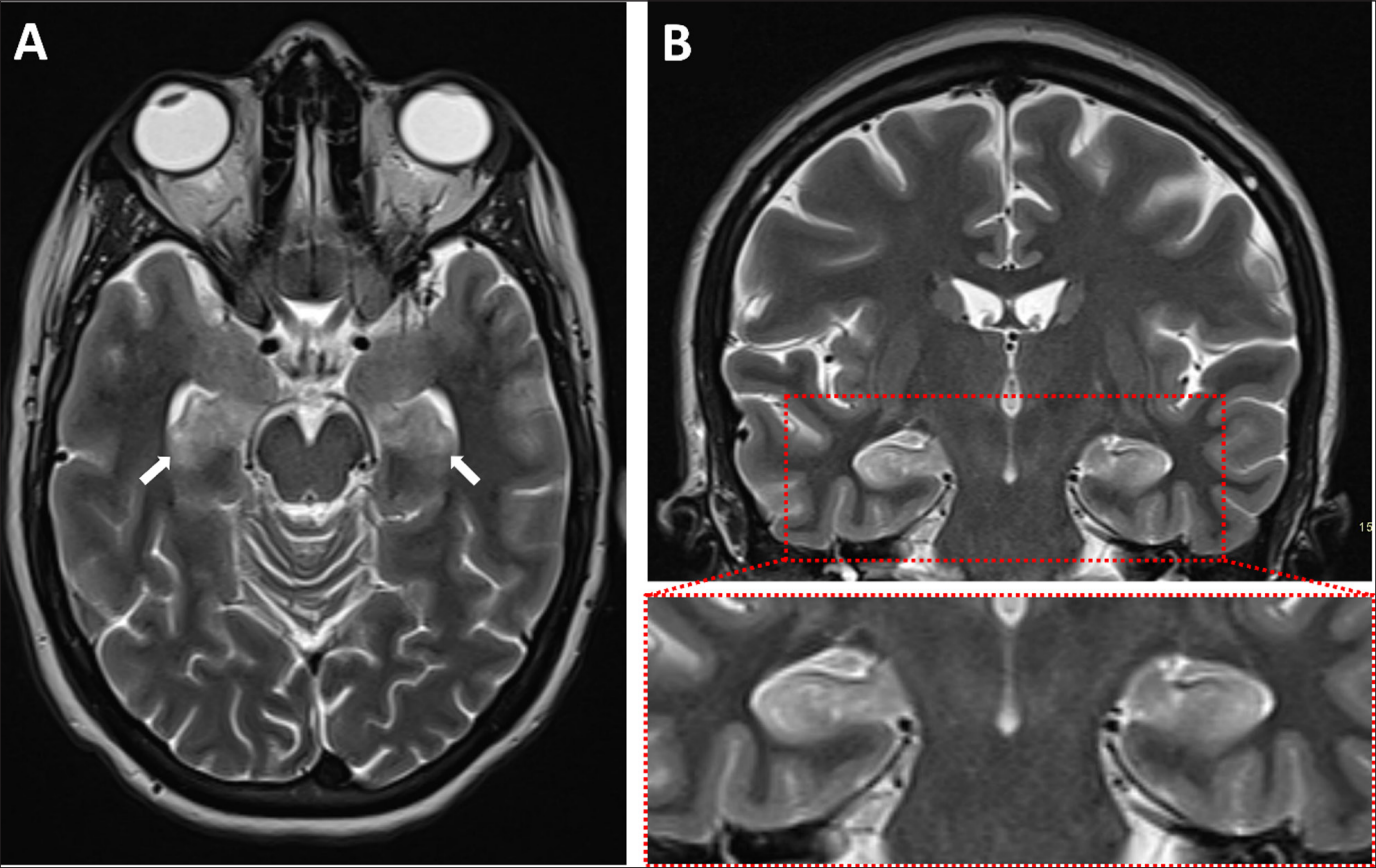


**Figure 3:**
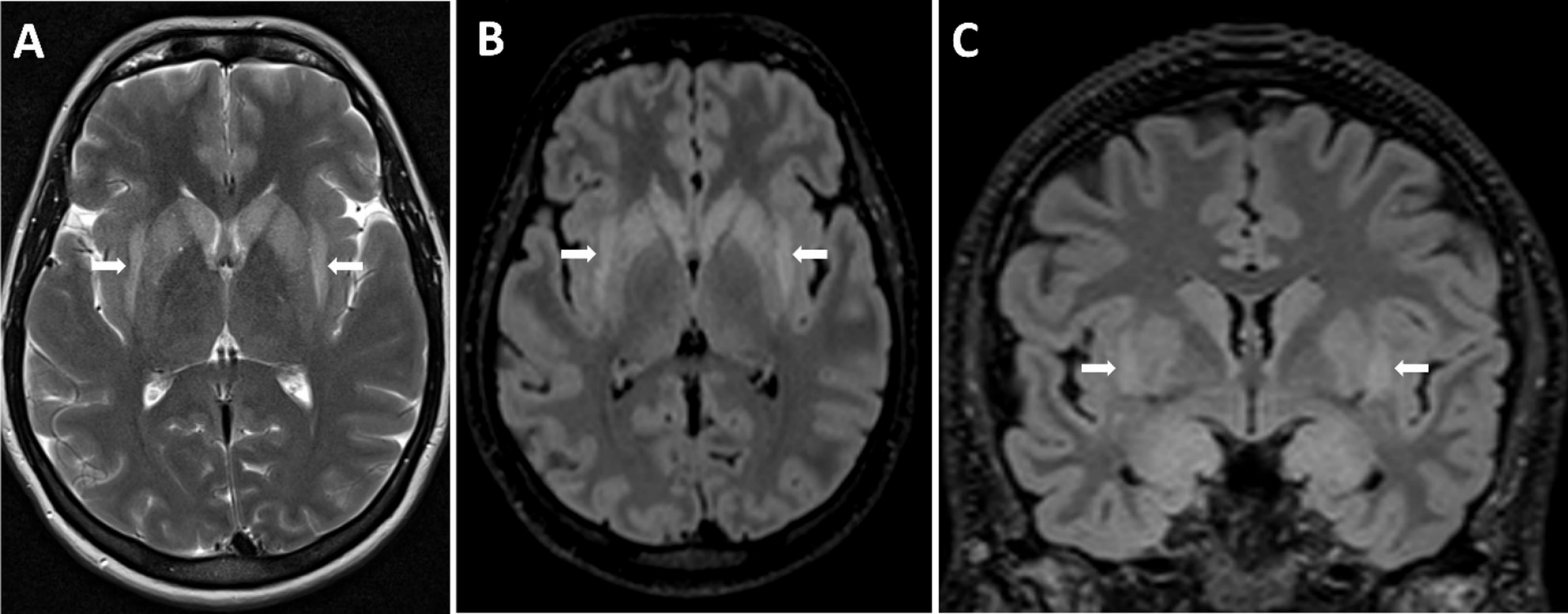


A 39-year-old woman who recently suffered an influenza-infection presented at the ER because of confusion, hallucinations, and progressive neurological decline. During her hospitalisation, she developed focal myoclonic epilepsy which transformed into status epilepticus. Magnetic resonance imaging (MRI) of the brain showed bilateral T2-hyperintense changes in the mesiotemporal structures on axial FLAIR-weighted images (Figure 1, arrows) and on axial and coronal T2-weighted images (Figure 2A–B respectively, arrows). Additionally, a bilateral T2-hyperintense claustrum was seen on axial T2 and axial and coronal FLAIR-weighted images (Figure 3A–C respectively, arrows). Lumbar puncture showed mild pleiocytosis and mildly elevated protein levels. Viral serological and PCR tests were negative. Clinical findings without detection of an acute seizure cause are compatible with *febrile infection-related epilepsy syndrome*.

## Comments

*Febrile infection-related epilepsy syndrome* (FIRES) is a subtype of *new-onset refractory status epilepticus* (NORSE). FIRES is a syndrome characterized by new refractory status epilepticus in a previously healthy (non-epileptic) patient who suffered a febrile illness two weeks to 24 hours prior to the seizure start. FIRES can be seen at any age, but most patients are school-aged children and young adults [1].

FIRES is a diagnosis of exclusion. Lumbar puncture shows mild pleiocytosis and mildly elevated protein levels in the majority of patients [1]. Infectious and autoimmune screens are usually negative, and no causative microorganism or antibody can be identified.

The role of imaging is mainly to rule out encephalitis or another structural seizure cause. The majority of (initial) imaging studies in FIRES are normal. When abnormalities are present, these often consist of T2-hyperintense changes in the mesiotemporal regions, with or without signal changes in the basal ganglia or peri-insular region [1]. Evolution to hippocampal sclerosis in the chronic phase is possible.

Another finding that was present in our patient, is the claustrum sign: bilateral T2-hyperintense signal changes in the claustrum. The claustrum sign is neither diagnostic nor specific for NORSE or FIRES however and has also been reported in other diseases such as acute necrotizing encephalopathy, COVID-19-related encephalopathy, and immune effector cell-associated neurotoxicity syndrome [1]. What all these entities have in common is a characterised cytokine storm, so the claustrum sign might be a radiological marker of cytokine-mediated neuro-inflammation.

NORSE and FIRES are associated with considerable morbidity and mortality. Most patients evolve to super-refractory status epilepticus and mortality rates are ~12% in children and up to 27% in adults. Functional outcome is poor, with half to two thirds of survivors having cognitive and functional impairment, and most patients having refractory epilepsy [1].

## References

[1] Mantoan Ritter L, Nashef L. New-onset refractory status epilepticus (NORSE). Practical Neurology. 2021; 21: 119–127. DOI: 10.1136/practneurol-2020-002534 33674412

